# Cognitive schemes and strategies in diagnostic and therapeutic decision making: a primer for trainees

**DOI:** 10.1007/s40037-013-0070-3

**Published:** 2013-07-11

**Authors:** Imad Salah Ahmed Hassan

**Affiliations:** Department of Medicine 1443, King Saud bin Abdulaziz University for Health Sciences and King Abdulaziz Medical City, P. O. Box 22490, Riyadh, 11426 Saudi Arabia

**Keywords:** Schemes, Decision-making, Novices, Experts

## Abstract

Unlike novices, expert clinicians develop refined schemes and strategies that predictably allow them to provide a better quality, prompt and less error-prone patient care input. Empowering novices with cognitive aids or mental schemes as early as possible in their clinical career may significantly improve their critical thinking, problem-solving and decision-making skills. These cognitive aids may also improve trainees’ use of evidence-based medicine in addition to reducing their diagnostic errors and improving their therapeutic care inputs.

## Introduction

Optimal problem-solving, clinical reasoning and rational decision-making are indispensable skills for quality care provision. These coupled with a comprehensive knowledge base are the two components of an ‘expert medical practitioner’. Cognitive conceptual deficiencies in decision-making have been shown to be an important cause of diagnostic errors, deficient therapeutic interventions and poor outcomes in both acute and ambulatory care settings [1–5]. Unlike novices, clinical experts tend to utilize ‘mental schemes’ for problem-solving, clinical reasoning and rational decision-making [6]. Research has confirmed that equipping trainees with the experts distinguishing, scheme-driven strategies significantly improves their decision-making skills, specifically in the diagnosis domain [7]. In essence, these schemes are knowledge and experience-based, cognitive aids that facilitate knowledge retrieval from the expert’s memory, thereby enhancing the practical instigation of a logical and organized problem-solving approach. It is anticipated that scheme-based cognitive training of novices and juniors will enhance their diagnostic problem-solving and decision-making abilities at an earlier stage in their career [6].

Generic cognitive aids or schemes presented in easy to recall, structured concept maps may thus serve as simple reminders to front-line staff, especially novices, on how to approach diagnostic and therapeutic uncertainties peculiar to their patients. It is generally believed that clinicians utilize two modes of reasoning for decision-making, namely System 1 and System 2 [8–10]. System 1 is a non-analytic, fast and intuitive one usually based on previous exposure whilst System 2 is an analytic, slow and rational mode acquired through structured training. Both are generally used interchangeably, yet System 1 is more error-prone. Cognitive aids used as ‘cognitive forcing strategies’ [11] should in principle facilitate and promote the use of System 2 in critical thinking and decision-making.

In this monograph, an approach for diagnostic and therapeutic decision-making using cognitive aids or schemes is presented. Cognitive aids, schemes and concept maps are used interchangeably. Hypothetical case scenarios are portrayed to assist in a better understanding of the concepts depicted in the monograph. Table 1 portrays the various steps or actions map in a patient encounter and its recommended cognitive schemes.

**Table 1:** Actions map for a patient encounter and their cognitive schemes

Step	Clinical action	Scheme/cognitive aid
1	Gather information (history and physical)	–
2	Propose a diagnosis	Pattern-recognition hypothetico-deductive strategies and smart heuristics, rule-out worst scenario, red flags, etc.
3	Differential diagnosis	Differential diagnosis cognitive aids: anatomical, physiological, pathological
4	Order tests (rationally)	Frugal heuristics probability assessment: test sensitivity, specificity and likelihood ratios
5	Confirm and comprehensively give a diagnostic label	Guideline-friendly bedside diagnosis, aetiology, severity (BESD)
6	Therapeutic interventions	Contextual, patient-centred therapeutic cognitive aid: site of care, symptomatic, supportive, specific and speciality referral (5S)
7	Prepare for discharge	Assess response to treatment (subjective and objective), criteria for discharge, timing of follow-up (ACT)

## Step 1: building knowledge and summarizing the problem

The first step in any clinical encounter is ‘information gathering’. This is achieved through history taking and physical examination. A skilled yet brief visual and auditory assessment of the patient allows the relatively experienced clinician to decide on the severity and seriousness of the presenting symptom. Once a complete and focused history and physical examination are completed, a vital step and an essential prerequisite before proceeding any further is to skilfully articulate a short summary of the clinical history and examination findings emphasizing only the positive and relevant features. The latter should additionally be phrased in conclusive technical medical terms, e.g. symptoms of lateral chest pain with coughing are qualified as pleuritic, red urine as haematuria, non-swollen, painful joints as arthralgia, stony dullness on examination as pleural effusion, enlarged spleen as splenomegaly, a single, swollen, painful joint as mono-arthritis, etc. Mastering this skill differentiates the novice from the expert and is generally conducive to better decision-making [12]. A structured, summary template for generic use is shown in Box 1.

**Box 1:** Summarizing the history and physical examination

Comprehensive but concise, text-book-like:
Must contain patient’s name, gender, age, ±occupation, ±nationality, ±racial/geographic origin, relevant past history/social history/family history, drug/allergic history, symptoms +duration—in technical terms, relevant physical signs in technical conclusive terms

## Step 2: making the diagnosis

The next step is making a bedside clinical diagnosis or a short list of a few differential diagnoses. This is probably the most crucial step in a patient encounter and the most error-prone [1, 2, 4]. Cognitive as well system errors contribute to patient harm and poorer outcomes [1, 2, 4]. As such, cognitive, individual or caregiver aids and strategies (as well as system interventions, see below) to enhance the trainees’ diagnostic accuracy and therapeutic interventions are indispensable [13, 14].

A four-phased scheme is depicted:

### Reaching a bedside clinical diagnosis using pattern recognition and hypothetico-deductive strategies [15]

Pattern recognition is the simplest and non-analytic ‘spot diagnosis’ of a clinical presentation usually based on classic visual clues or specific test finding. For example, the rash of herpes zoster, the facies of a patient with acromegaly and the electrocardiogram findings of an acute myocardial infarction. Another pattern-recognition strategy is achieved through heuristics [16, 17]. Heuristics are mostly history-based, expert-employed, pattern-recognizing ‘rules of thumb’ or short-cut decision strategies that rely on a small fraction of the gathered information (relevant or trustworthy predictors) for considering a diagnosis. For example, a middle-aged smoker with central chest pain radiating to his left upper limb will automatically be labelled as having an acute coronary syndrome. Similarly, a postoperative patient with a single swollen leg, shortness of breath and haemoptysis will be labelled as suffering from pulmonary thromboembolism and a 12-year-old with a right-iliac fossa pain that started para-umbilically and is associated with anorexia and vomiting will be given the diagnosis of acute appendicitis. Although both visual and history-based pattern recognition strategies are fast decision/diagnostic strategies, heuristic, pattern recognition is of lower fidelity and reliability than visual, pattern recognition spot diagnosis and is thus more error-prone [16, 17].

However, many clinical encounters and diagnostic challenges are primarily unravelled using another strategy: the hypothetico-deductive strategy [15]. Clinicians utilize clinical and epidemiological clues from the information gathered by history-taking and possibly substantiated by physical examination to arrive at a single diagnosis or a short-list of differential diagnosis. As mentioned above, this is a critical and error-prone stage for novices [1, 4]. Skilled experts revert to at least two other strategies to solve the diagnostic puzzle whilst excluding immediate life-threatening or ‘not-to-miss’ diagnoses: ‘red flags’ and ‘rule out the worst scenario’ (ROWS) [18].

ROWS and red flags are strategies that assist the clinician to avoid missing the most serious of the possible differential diagnoses. For example, the expert will automatically enquire, examine and investigate for the more serious causes of central chest pain such as acute coronary syndrome and aortic dissection rather than for the other less serious causes such as oesophageal spasm. Similarly, meningitis and intracranial vascular events will be the primary concerns for the expert interviewing a patient with headache. Red flags for the latter scenario (acute meningitis) may include symptoms such as fever and photophobia and signs such as neck stiffness and change in sensorium. Checklists of red flags may be utilized by the novice to safeguard against missing serious problems.

A simple heuristic that helps to narrow the differential diagnosis is trying to categorize the disease as secondary to one organ/system involvement or multi-systemic. A patient with fever and primarily respiratory-associated symptomatology points to a respiratory system pathology while the presence of symptoms related to several organ systems point to a multi-system disease.

### Constructing a differential diagnosis

An important and well-recognized cause of diagnostic errors is failing to consider alternative diagnoses [3, 5]. This is inherent to fully relying on heuristics for reaching a clinical diagnosis [16, 17]. Heuristics as such are obviously error-prone. Trainees must be equipped with simple concept maps or cognitive aids to seamlessly construct a list of possible differential diagnoses [7]. These ‘schemes’ guide the trainee in constructing a hypothesis-driven [19, 20], focused, rational, history taking, examination and investigation plan. Three cognitive aids are depicted in Table 2. The differential diagnosis of pain and swellings is generally anatomical. Physiological differential diagnosis listing is especially applicable to two medical problems, namely shock and thrombosis. All differential diagnosis listings may, however, be easily structured along the two pathological or aetiopathological entities of: congenital/hereditary or acquired. The latter may be sub-classified into 10 categories: traumatic, infective, inflammatory/autoimmune, vascular/degenerative, neoplastic/para-neoplastic, metabolic/endocrine, drug-induced/poisoning, deficiency diseases, psychogenic and idiopathic/cryptogenic.

**Table 2:** Differential diagnosis cognitive aids

Anatomical differential diagnosis	Physiological differential diagnosis	Aetiopathological differential diagnosis
*Pain syndromes* e.g. central chest pain may be categorized as arising from the heart, aorta, oesophagus, chest wall etc.	*Shock* this may be hypovolaemic, distributive, obstructive or cardiogenic	Congenital or hereditary
*Swellings* e.g. a neck swelling differential diagnosis will include the thyroid, lymph nodes, vascular, skin etc.	*Thrombosis* this may be related to a vessel wall pathology, blood constituents or flow rate	Acquired
		1. Traumatic
		2. Infective: viral, bacterial etc.
		3. Inflammatory/auto-immune
		4. Vascular/degenerative
		5. Neoplastic/para-neoplastic
		6. Metabolic/endocrine
		7. Drug-induced/poisoning
		8. Deficiency diseases
		9. Psychogenic
		10. Idiopathic/cryptogenic

### Rationally ordering a test or tests based on a practical ‘fast-and-frugal’ probability scoring

One major difficulty trainees’ exhibit after a patient encounter is coming-up with a clinical probability for the possible diagnosis or differential diagnoses. Probability estimation (based on the presence of risk factors and clinical findings) is crucial for appropriate and rational diagnostic test ordering. An appropriate and practical probability calculation or assessment methodology is the use of specific clinical scoring or decision support tools such as the Well’s criteria for assessing the probability of pulmonary thromboembolism. However, a more generic tool based on the presence of a strong risk factor(s) for the problem or diagnosis and clinical absence of alternative possibilities may be used for probability assessment. Thus the presence of strong risk factor(s) for the problem or diagnosis coupled with the absence of other significant competing differential diagnosis-supporting findings qualifies the presumed diagnosis as high probability. On the other hand, if only one of the two statements is true, the diagnostic probability is intermediate and if both are negative, the probability is considered low. This ‘frugal heuristic’ [21, 22] which is defined as the ability to reach a good probability assessment with limited information, is thus fast and easily applicable. For example, a breast lump in a 30-year-old is unlikely to be cancerous. However, the presence of strong risk factor such as a family history or hormone replacement therapy use and clinical absence of symptoms and signs of infection or history of trauma, breast feeding etc., makes cancer a high probability.

Tests are then ordered based on their sensitivity and specificity for the possible diagnosis [23, 24]. A composite of a test’s sensitivity and specificity is the likelihood ratio. Definitions of sensitivity, specificity and likelihood ratios are shown in Table 3. The rules for appropriate ordering are based on the clinician’s probability assessment. Tests with high specificity (usually more expensive) are appropriate for high and intermediate-probability assessments, especially when the considered diagnosis is life-threatening such as spiral computerized tomographic pulmonary angiography for a high probability embolism. On the other hand, highly sensitive tests (usually less expensive) are appropriate for low probability patients and for screening such as d-dimer testing for patients with low probability for pulmonary embolism, Tuberculin test, or faecal occult blood testing. The mnemonics for these are SpIn: highly specific tests are useful for ruling-in the diagnosis when positive and SnOut: highly sensitive tests are useful for ruling-out the diagnosis when negative. As such, highly specific tests are useful when positive and highly sensitive tests are negative. It is worth noting, however, that highly sensitive tests may also help in prognostication and assessing response to treatment when they are indeed positive. Brain natriuretic peptide is a highly sensitive test. When negative, it almost completely rules out left ventricular failure as a cause of pulmonary oedema [25]. However, the higher the reading, the worse the prognosis [25]. Reduction of levels to normal confirms improvement with treatment [25].

**Table 3:** Sensitivity, specificity and likelihood ratios: definitions and examples

Sensitivity	*Example* in a group of 100 patients *with bacterial pneumonia*, 80 had a raised C-reactive protein CRP: the sensitivity of CRP for diagnosing bacterial pneumonia is thus 80 %
How often is the test result correct for persons in whom the disease is known to be present?	
Sensitivity—the proportion of people *with* disease who have a positive test	
Specificity	*Example* in a group of 100 patients *without pneumonia*, 10 had a raised C-reactive protein CRP: the specificity of CRP for correctly excluding pneumonia is thus 90 %
How often is the test result correct for persons in whom the disease is known to be absent?	
Specificity—the proportion of people *without* the disease who have a negative test	
Likelihood ratio	*Example* A raised jugular venous pressure (JVP) in a patient with a history suggestive of congestive heart failure (CHF) has a positive likelihood ratio of 5.8 and a negative ratio of 0.66. Thus the presence of a raised JVP *rules-in* the diagnosis of CHF. Its absence is not as useful in *ruling it out*
The likelihood that a given test result would be expected in a patient with the target disorder compared with the likelihood that the same result would be expected in a patient without that disorder.	
In general, a positive likelihood ratio of *4 or more* is useful in *ruling-in* the target disorder. A negative likelihood ratio of <0.3 is useful in *ruling-out* the target disorder	

A comprehensive knowledge of the sensitivity, specificity and likelihood ratios of commonly used tests is therefore essential.

### Appropriate diagnostic labelling: the BESD diagnosis cognitive aid

The bedside clinical diagnosis, a etiological cause and severity score diagnostic labelling (BESD) concept map for comprehensive diagnostic labelling has been described previously [26]. Trainees should be able to comprehensively provide a full label that explicitly portrays the three essential domains of diagnosis: bedside clinical diagnosis, aetiology or precipitant, and severity. Guidelines unambiguously recommend severity scoring for many clinical conditions, for example for community-acquired pneumonia, bronchial asthma, acute pancreatitis and stroke. Commonly, trainees have a tendency to incompletely provide a diagnostic label for their patients. For example, labelling a patient with community-acquired pneumonia as such without paying attention to the possible aetiology, e.g. influenza A or bacterial pneumonia or severity e.g. the CURB-65 score, may inevitably result in lower quality and deficient care and poorer outcomes.

The practical use of the four phases above in diagnosing a patient may be conducive to a reduction in diagnostic errors, improved and rational use of diagnostic tests and better guideline implementation.

## Step 3: immediate therapeutic interventions: the 5S cognitive aid

Similar to the BESD model, the 5S concept map has also been described previously [26]. The 5S therapeutic concept map (site of care, symptomatic treatment, supportive care, specific care, speciality referral) is considered a simple cognitive aid that will assist the practising physician (especially front-line staff in the emergency room) in constructing an evidence-based, patient-centred, timely and comprehensive therapeutic plan. Guidelines unambiguously dictate sites of care for specific disease severity scores or categories, e.g. in a patient with diabetic ketoacidosis and significant hypokalaemia or hyperosmolarity. Prompt provision of symptomatic treatment is important as it directly alleviates patient discomfort. Symptom relief is regrettably not regularly ordered by medical staff. An excellent example is the poor use of analgesics in the acute care setting, referred to as oligoanalgesia. Similarly, prompt use of supportive care to improve physiological derangements before damage becomes irreversible and until the precipitant is brought under control by its specific intervention may be life-saving, e.g. oxygen therapy in hypoxic patients, intravenous fluids in patients with hypovolaemic shock, or sodium bicarbonate in severely acidotic patients. Correctly providing specific care to treat the primary cause or aetiology is a fundamental step in patient care. Guidelines recommend early speciality or sub-speciality referral for specific acute illnesses, e.g. patients with acute coronary syndromes or significant upper gastrointestinal haemorrhage and associated co-morbidities need to be referred early to their respective specialities.

## Step 4: the ACT cognitive aid: assessment of response to treatment, criteria for discharge and timing of follow-up

It is critical and imperative that once a diagnosis is reached and a therapeutic intervention is instigated, at least three other practical actions are undertaken. Firstly, the assessment of response to treatment: a satisfactory response to one’s therapeutic intervention is a solid proof that the diagnosis was correct and appropriate. Usually, assessment of response is based on both subjective and objective measures. The latter include either clinical criteria such as fever, vital signs etc. or laboratory and imaging and other investigations. Failing to internalize clear and solid criteria for home discharge or other patient disposition areas results in unnecessary and longer hospital stays. The majority of patients who are discharged from hospital will require follow-up visits. These are required for both disease and drug monitoring. Appropriateness and timeliness of such visits may assist in reducing the readmission rates.

## Final remarks

Apart from individual or trainee-directed cognitive interventions, system-based interventions for reducing diagnostic and therapeutic errors and deficiencies must similarly be put in place. Such system tools include curricula for regular training and assessment of staff in decision-making skills and bias recognition, use of reminders such as clinical pathways, protocols, order sets, checklists, use of computerized decision support tools, mechanisms for error detection and rectification and a general improvement in knowledge access by all staff [12, 13, 27, 28].

**Fig. 1:**
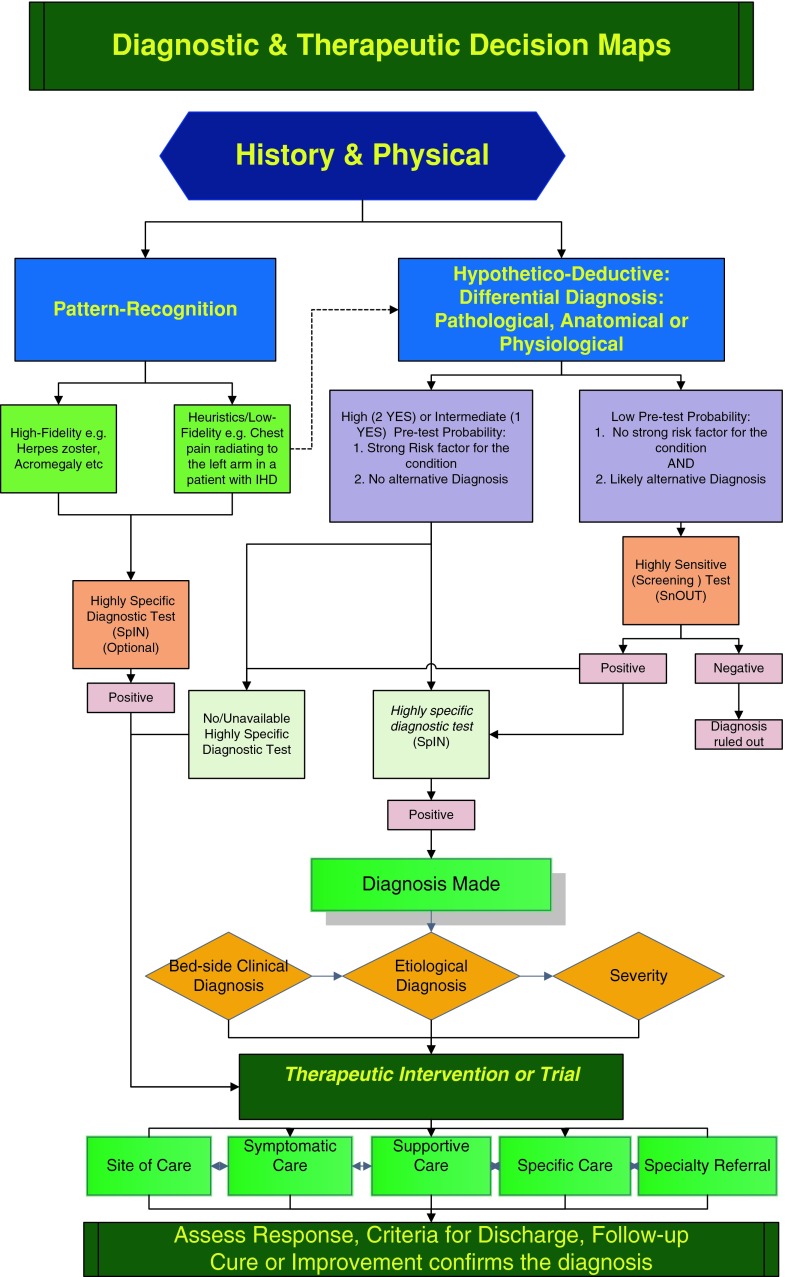
Diagnosis and therapy cognitive maps

Table 4 portrays a case scenario illustrating the use of the expert summary, BESD, pathological differential diagnosis and 5S therapeutic interventions schemes. Figure 1 is a graphic summary of approaching a diagnostic challenge and the immediate therapeutic interventions and further care inputs.

**Table 4:** A case scenario illustrating the use of the ‘technical’ expert summary, BESD, pathological differential diagnosis and 5S therapeutic interventions

• 67-year-old male	
• Bird/pigeon breeder, smoker	
• 3-day history of fever, cough with yellow sputum, left stabbing chest pain that is worse with breathing and coughing and breathlessness	
• Clinically, breathless, cyanosed, disoriented to time, person and place,	
Temperature 39.1 °C	
• BP 86/50 mmHg, RR 32/min, bilateral coarse crepitations, bronchial breathing left lower zone	
• Chest X-ray: left basal consolidation	
Summary	
67-year-old, smoker and bird-breeder presenting with a 3-day history of productive cough, dyspnoea and left pleuritic chest pains	
Clinically confused, cyanosed, febrile, tachypnoiec and hypotensive with signs of left lower zone consolidation	
1. Bedside-clinical diagnosis	Community acquired pneumonia with septic shock
2. Cause/precipitant	Chlamydia psittaci
	Aetio-pathological differential diagnosis
	Other Infections: e.g. avian flu, cryptococcal infection
	Inflammatory e.g. collagenosis, allergic alveolitis
	Vascular e.g. pulmonary embolism
	Neoplastic, drug-induced etc.
3. Severity	Life-threatening (CURB-65 = 4)
4. Site of care	ICU
5. Symptomatic	Analgesia, anti-pyretic
6. Supportive	Oxygen, intravenous fluids
7. Specific	Antibiotics
8. Speciality referral	Intensive therapy unit, pulmonary service

## Essentials

1. Cognitive conceptual deficiencies in decision-making are recognized as an important cause of poor patient care.
2. Unlike novices, experts develop robust and complex schemes that facilitate the provision of higher-quality and time-efficient care inputs.
3. Empowering trainees with explicit, generic schemes of care early in their clinical career may hasten their novice to expert critical thinking, problem-solving and decision-making skills acquisition as well as improve their use of evidence-based medicine.
